# Impacto de um Programa de Redução do Estresse, Meditação e *Mindfulness* em Pacientes com Insuficiência Cardíaca Crônica: Um Ensaio Clínico Randomizado

**DOI:** 10.36660/abc.20220768

**Published:** 2023-10-16

**Authors:** Vaisnava Nogueira Cavalcante, Evandro Tinoco Mesquita, Ana Carla Dantas Cavalcanti, Jacqueline Sampaio dos Santos Miranda, Paola Pugian Jardim, Glaucio Martins da Silva Bandeira, Lais Marcelle Rufino Guimarães, Isabella Christina Diniz de Lemos Venâncio, Nathalia Manoela Condeixa Correa, Angela Maria Rodrigues Dantas, João Carlos Tress, Ana Catarina Romano, Fabiana Bergamin Muccillo, Marina Einstoss Barbosa Siqueira, Glaucia Cristina Andrade Vieira

**Affiliations:** 1 Universidade Federal Fluminense Niterói RJ Brasil Universidade Federal Fluminense, Niterói, RJ – Brasil; 2 Universidade Federal Fluminense Departamento de Fundamentos de Enfermagem e Administração Rio de Janeiro RJ Brasil Universidade Federal Fluminense - Departamento de Fundamentos de Enfermagem e Administração, Rio de Janeiro, RJ – Brasil; 3 Instituto Nacional de Cardiologia Rio de Janeiro RJ Brasil Instituto Nacional de Cardiologia, Rio de Janeiro, RJ – Brasil; 4 Complexo Hospitalar de Niterói Niterói RJ Brasil Complexo Hospitalar de Niterói (CHN), Niterói, RJ – Brasil

**Keywords:** Estresse Psicológico, Medicina Comportamental, Mindfulness, Meditação, Insuficiência Cardíaca

## Abstract

**Fundamento:**

A Insuficiência Cardíaca é um importante problema de saúde pública, que leva à alta carga de sintomas físicos e psicológicos, apesar da terapia otimizada.

**Objetivo:**

Avaliar primariamente o impacto de um Programa de Redução de Estresse, Meditação e Atenção plena na redução do estresse de pacientes com Insuficiência Cardíaca.

**Métodos:**

Ensaio clínico randomizado e controlado que avaliou o impacto de um programa de redução de estresse comparado ao atendimento multidisciplinar convencional, em dois centros especializados no Brasil. O período de coleta de dados ocorreu entre abril e outubro de 2019. Um total de 38 pacientes foram alocados nos grupos de intervenção ou controle. A intervenção aconteceu ao longo de 8 semanas. O protocolo consistiu na avaliação das escalas de estresse percebido, depressão, qualidade de vida, ansiedade, atenção plena, qualidade do sono, teste de 6 minutos de caminhada e biomarcadores por um grupo cego, considerando um p-valor <0,05 como estatisticamente significativo.

**Resultados:**

A intervenção resultou em redução significativa no estresse percebido de 22,8 ± 4,3 para 14,3 ± 3,8 pontos na escala de estresse percebido no grupo de intervenção vs. 23,9 ± 4,3 para 25,8 ± 5,4 no grupo controle (p-valor<0,001). Foi observada melhora significativa na qualidade de vida (p-valor=0,013), atenção plena (p-valor=0,041), qualidade do sono (p-valor<0,001) e no teste de 6 minutos de caminhada (p-valor=0,004) no grupo sob intervenção comparado com o controle.

**Conclusão:**

O Programa de Redução de Estresse, Meditação e Atenção plena reduziu efetivamente o estresse percebido e melhorou desfechos clínicos em pacientes com Insuficiência Cardíaca.

## Figura Central

**Figure:**
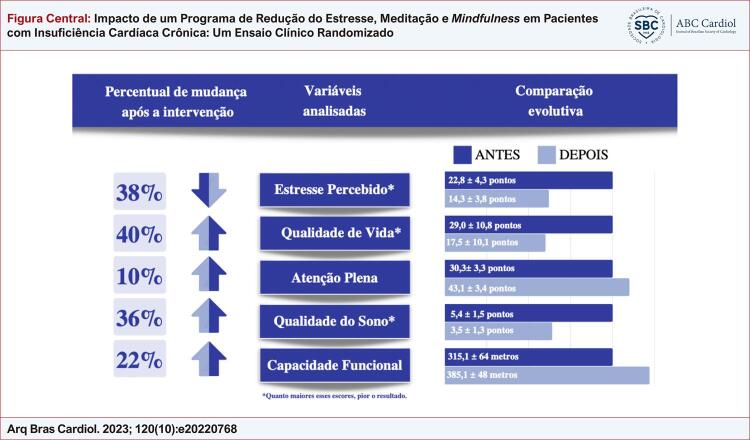
: Impacto de um Programa de Redução do Estresse, Meditação e Mindfulness em Pacientes com Insuficiência Cardíaca Crônica: Um Ensaio Clínico Randomizado

Resumo dos principais resultados do Programa de Redução do Estresse, Meditação e Atenção Plena. As variáveis estresse percebido, qualidade de vida, atenção plena e qualidade do sono foram medidas em pontos dos respectivos escores das escalas. A variável capacidade funcional foi medida em metros.

## Introdução

A insuficiência cardíaca (IC) é um importante problema de saúde pública que afeta 26 milhões de pessoas no mundo, com projeção de aumento em sua prevalência com o envelhecimento populacional e sobrevida prolongada.^1,2^ Ela é responsável por sobrecarga do sistema de saúde, pelas altas taxas de morbidade, mortalidade, hospitalização e custos.^3,4^

Estudos apontam altos índices de transtornos emocionais em pacientes com IC, o que piora sua morbidade, qualidade de vida e mortalidade.^5,6^O estresse psicossocial e seus componentes estão associados a maior ativação neuro-hormonal a desfechos desfavoráveis.^7-10^

Pacientes com IC convivem com elevado estresse psicossocial e existencial, explicado pela doença progressiva, limitante, que altera o status de trabalho e o convívio social e impacta suas qualidades de vida.^10^

Uma coorte prospectiva avaliou 6.985 indivíduos e demostrou que o baixo senso de propósito está significativamente associado à mortalidade por todas as causas. Isso mostra a necessidade de pesquisas que avaliem intervenções que promovam uma vida com significado para melhora de desfechos em saúde.^11^

Diversas evidências científicas sobre as interações cérebro-coração têm levado ao crescimento de pesquisas na cardiologia comportamental.^12^ Estudos exemplificaram a conexão entre condições de estresse psicossocial em humanos e aumento da atividade pro-inflamatória.^13-16^A indução do estresse leva à ativação do sistema nervoso autonômico (SNA) e ao aumento da produção de citocinas inflamatórias.^17^

A IC, por sua vez, é caracterizada por um estado inflamatório crônico e pelo desequilíbrio do SNA, o que contribui para a sua progressão.^18^ A associação entre quadros depressivos e IC poderia, assim, contribuir para o aumento do risco de mortalidade nestes pacientes.^19^

Tem sido demostrado que, por meio das emoções e pensamentos, o SNA tem um papel relevante na conexão cérebro-coração.^12^ Ao atuarem por meio do SNA, as práticas mente-corpo podem beneficiar o funcionamento endotelial, neuroendócrino e imunológico.^20-22^

A atenção plena (*mindfulness*) é definida como capacidade de prestar atenção no momento presente, intencionalmente e sem julgamento.^23^ Intervenções envolvendo a prática de atenção plena são efetivas na redução de depressão, atividade simpática, ansiedade, estresse psicossocial, dor, melhora de capacidade funcional e qualidade de vida.^24-29^ Níveis elevados de atenção plena estão relacionados a menores níveis de ansiedade em pacientes com IC.^30^

Poucos estudos foram desenvolvidos avaliando o impacto de intervenções baseadas em atenção plena nos pacientes com IC. Discute-se assim, a necessidade de realização de mais estudos com rigor metodológico e que avaliem o impacto destas intervenções no estresse percebido, na capacidade funcional bem como nos níveis de atenção plena destes indivíduos.^31^

Desta forma, o presente estudo teve como objetivo primário avaliar o impacto do Programa de Redução do Estresse, Meditação e Atenção plena (P.R.E.M.A.) no estresse percebido de pacientes portadores de IC em centros especializados. Escores de ansiedade, depressão, atenção plena, qualidade de vida, qualidade do sono, além de capacidade funcional (teste de 6 minutos de caminhada), painel inflamatório incluindo velocidade de hemossedimentação (VHS) e proteína c-reativa (PCR) e painel neuro-hormonal (cortisol e NT -proBNP) foram avaliados secundariamente.

## Métodos

### Desenho do estudo

Ensaio clínico randomizado realizado em quatro grupos paralelos e distintos. Os grupos da intervenção (I-CICCV e I-INC) participaram de um programa de 8 semanas de redução do estresse. Os grupos controles (C-CICCV e C-INC) receberam o atendimento multidisciplinar convencional da Clínica de Insuficiência Cardíaca Coração Valente (CICCV)/UFF e do departamento de Insuficiência Cardíaca e Transplante do Instituto Nacional de Cardiologia (INC), de onde os pacientes foram recrutados.

### Participantes

Foram incluídos pacientes ambulatoriais, acima de 18 anos, com diagnóstico de IC pelos critérios Framingham e/ou Boston; classificação da NYHA I-II; Mini Mental > 16, aderentes a pelo menos 80% dos encontros; alfabetizados ou com acompanhante alfabetizado; concordantes em participar das atividades propostas e em responder aos questionários de avaliação; que possuíssem dispositivo de som para ouvir os áudios com as práticas diárias.

Foram excluídos aqueles com participação em outro estudo/terapia com intervenção mente-corpo no último mês; com descompensação clínica recente com internação hospitalar ou alteração na prescrição medicamentosa no último mês; com cirurgia ou intervenção percutânea planejada para o próximo ano; com história de síndrome coronariana aguda ou infarto agudo do miocárdio nos últimos dois meses; com valvulopatia clinicamente significativa; com miocardite aguda; portadores de doenças osteo musculares que impedissem a realização de provas funcionais.

### Intervenção

O Programa de Redução do Estresse, Meditação e Atenção plena (P.R.E.M.A.) é baseado no tripé: ferramentas de *coping* (enfrentamento), de atenção plena e de *dharma* (termo sânscrito para propósito de vida). As sessões ocorreram ao longo de 8 encontros em grupo semanais de 2 horas de duração, que constavam de 4 momentos:

1. Introdução.
2. Apresentação do tema.
3. Questões para autoconsciência.
4. Prática guiada.

Os temas abordados foram: o estresse, a mente, resiliência, autorregulação emocional, aceitação, autoeficácia, gratidão, conexão social e propósito. Em cada sessão foi abordada uma ferramenta de enfrentamento relacionada ao tema.

As práticas de atenção plena abordadas ao longo das sessões foram atenção à respiração, comer consciente, inventário de atenção às emoções negativas e positivas, meditação da compaixão, caminhar consciente, meditação da reciprocidade.

As sessões ocorreram entre maio e junho de 2019 nas instalações da CICCV (para I-CICCV) e do INC (para I-INC). O período de coleta de dados ocorreu entre abril e outubro de 2019. O programa foi facilitado pela pesquisadora principal do estudo, cardiologista com 23 anos de experiência em meditação e *bhakti yoga, internato* em hospital com foco em terapias mente-corpo-espírito (Bhaktivedanta Hospital – Mumbai/Índia), treinamento nas técnicas SMART® (Stress Management and Resilience Training) e Mindfulness pela Case Western Reserve University e certificação em yoga pela VVY School.

Materiais de apoio como apostila e CD de áudio para prática diária de 45 minutos foram fornecidos. A adesão às tarefas foi observada pelos relatos dos pacientes, diário de meditação e por telemonitoramento.

O desfecho primário avaliado foi o estresse percebido e os desfechos secundários foram os escores de ansiedade, depressão, atenção plena, qualidade de vida, qualidade do sono, capacidade funcional (T6MC), painel inflamatório (VHS, PCR) e neuro-hormonal (cortisol e NT-proBNP).

### Tamanho da amostra

A amostra foi calculada com base em estudo prévio que avaliou o desfecho “estresse percebido”, no qual se utilizaram os seguintes valores dos escores: antes da intervenção (grupo controle=19,1/DP±8,9; grupo intervenção=25,8/DP±4,6) e depois da intervenção (grupo controle=20,5/DP±10,3; grupo intervenção=20,2/DP±5,6).^32^ A amostra final foi de 36 pacientes, 18 para o grupo da intervenção e 18 para o grupo controle, após considerar diferença de 0,3 pontos, confiança 95%, perda 20% e poder de 80%.

### Randomização

A randomização foi realizada por meio do website http://randomization.com considerando 20% de perda. Um profissional externo ao grupo da pesquisa foi responsável pela lista gerada para alocação dos pacientes. A proporção de alocação foi de 1:1 para os grupos de intervenção ou controle.

Por se tratar de intervenção comportamental, não foi possível o cegamento do facilitador ou pacientes. Porém, houve cegamento em relação à avaliação, feita por um grupo de investigadores responsáveis pela coleta de dados e armazenamento dos resultados dos questionários, teste funcional e biomarcadores.

A equipe de pesquisa foi constituída de grupo de avaliação, grupo de intervenção, grupo de acompanhamento do controle e grupo de randomização.

### Avaliação

A avaliação pré-intervenção ocorreu uma semana antes do início da intervenção e constou de consulta presencial com equipe multiprofissional das clínicas especializadas.

Na consulta presencial o paciente respondeu aos instrumentos de coleta de dados: 1) Mini exame do estado mental (Mini Mental); 2) Escala de estresse percebido, 14 itens (PSS-14); 3) Inventário de depressão de Beck, segunda versão (BDI-II); 4) Questionário de qualidade de vida de Minnesota (MLHFQ); Inventário de ansiedade traço-estado (IDATE) – IDATE-T (traço) e IDATE-E (estado); Índice de qualidade do sono de Pittsburgh (PSQI); e a versão brasileira do Inventário Freiburg de Mindfulness (FMI-Br).

O PSS-14 é uma escala de autorrelato para mensuração do estresse percebido, composta por 14 itens com escores que vão de 0 a 56. Quanto mais elevados os escores, piores os níveis de estresse. O IDATE traço ou estado também é uma escala de autorrelato que possui 20 itens, com escores que vão de 20 a 80. Altas pontuações em suas respectivas escalas significam maior traço ou estado de ansiedade. O BDI-II é uma escala de autoavaliação composta de 21 itens com escores que variam de 0 a 63. Quanto mais elevado o escore, mais grave a depressão.

O MLHFQ é uma escala de 21 itens com escore global que varia de 0 a 105 e escore mais baixo reflete melhor qualidade de vida. O PSQI avalia 7 componentes do sono com escore global que varia de 0 a 21. Quanto maior a pontuação, pior a qualidade do sono. O FMI-Br identifica a frequência com que a pessoa vivencia comportamentos relacionados à atenção plena. A pontuação varia de 14 a 56 pontos. Quanto maior a pontuação obtida, maior é a percepção de atenção plena.

Neste dia, também foi realizado o T6MC por profissionais de fisioterapia que utilizaram a diretriz da *American Thoracic Society*, bem como coleta de sangue para análise de painel inflamatório e neuro-hormonal pelo laboratório do INC.

A avaliação pós-intervenção aconteceu uma semana após o término da intervenção, incluindo novamente os questionários citados, coleta de sangue para as provas laboratoriais realizadas inicialmente, bem como o T6MC. O grupo controle recebeu atendimento multidisciplinar habitual das clínicas de IC. Por se tratar de centros de pesquisas especializadas, os pacientes tiveram garantia de continuidade no atendimento.

Foi realizado estudo piloto com 3 pacientes da clínica CICCV externos ao estudo, antes da intervenção propriamente dita, objetivando avaliar a factibilidade do programa e realizar ajustes na intervenção.

### Análise estatística

Utilizou-se o *software* SPSS versão 24 para confecção dos gráficos e análises dos dados. Para resumir os dados utilizou-se a média acrescida de seu intervalo de confiança de 95%. Os resumos dos dados foram dispostos em gráficos que apresentam as combinações entre as duas categorias do tempo e as duas categorias do grupo, totalizando quatro barras.

As variáveis contínuas foram descritas por medidas de média e desvio padrão. As variáveis categóricas foram descritas com frequências simples e percentual. O teste de normalidade para confirmação da distribuição normal foi o Shapiro-Wilk.

Aplicou-se a metodologia modelo linear generalizado (MLG) para medidas repetidas, para verificação dos efeitos de interação, tempo e grupo. Havendo resultados significantes nos efeitos, aplicou-se pós teste utilizando a correção via Bonferroni para comparar quais médias diferem entre si e adotou-se p-valor <0,05 como nível de significância no estudo.

### Aspectos éticos

Esta pesquisa foi aprovada pelo Comitê de Ética e Pesquisa da Faculdade de Medicina do Hospital Universitário Antônio Pedro, parecer 3.224.212, bem como pelo Comitê de Ética e Pesquisa do INC, parecer 3.339.599, assim como foi registrada no Registro Brasileiro de Ensaios Clínicos, sob número RBR-7pzcyk.

## Resultados

**Figura 1:**
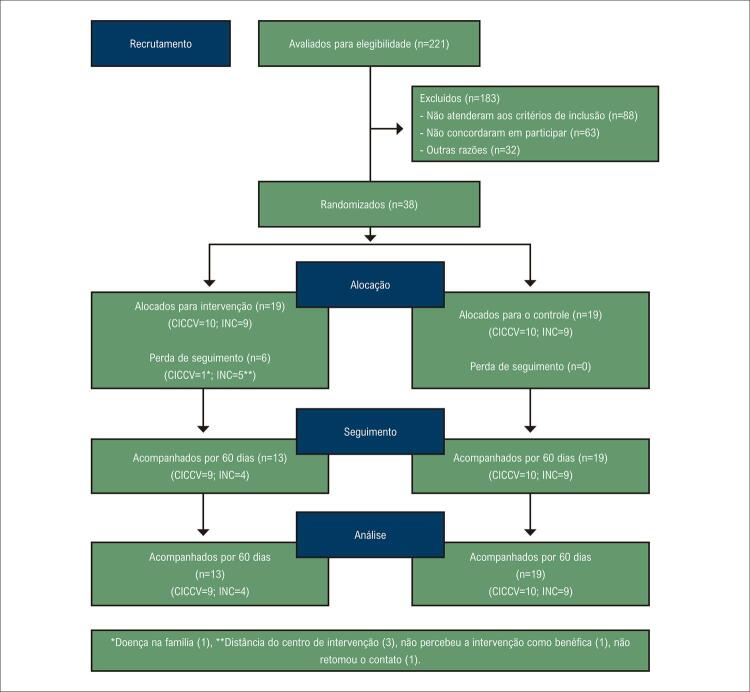
– Fluxograma do processo de seleção de participantes para o ensaio clínico randomizado.

Foram avaliados 221 pacientes para inclusão no estudo por meio de pesquisa em prontuário médico. Destes, 133 eram elegíveis, sendo 101 pacientes contactados e os demais não abordados por não possuírem contato telefônico atualizado. O total de 38 pacientes concordaram em participar e foram randomizados para os grupos de intervenção e controle. Dos 38, 32 pacientes completaram os protocolos de avaliação, sendo 13 do grupo intervenção (I-CICCV=9 e I-INC=4) e 19 do grupo controle (C-CICCV=10 e C-INC=9). A Figura 1 ilustra a seleção e recrutamento dos pacientes.

A amostra foi composta predominantemente por homens, autodeclarados de raça parda, NYHA II, com ensino médio completo e portadores de insuficiência cardíaca com fração de ejeção reduzida. A Tabela 1 ilustra as características sociodemográficas de base da amostra inicial de 38 pacientes:

**Tabela 1:** – Características sociodemográficas e clínicas dos grupos intervenção (n=19) x controle (n=19). Brasil, 2019

Variáveis	Intervenção (n=19)	Controle (n=19)
Idade (anos)	58,0±12,8^*^	54,3±9,8^*^
**Sexo**		
Masculino	12 (63,2)	13 (68,4)
Feminino	7 (36,8)	6 (31,6)
**Raça autodeclarada**		
Branco	6 (31,6)	3 (15,8)
Negro	4 (21,1)	4 (21,1)
Pardo	9 (47,4)	12 (63,2)
**Classe da NYHA**		
I	7 (36,8)	5 (26,3)
II	12 (63,2)	14 (73,7)
**Escolaridade Completa**		
Ensino fundamental	10 (52,6)	4 (21,1)
Ensino médio	9 (47,4)	14 (73,7)
Pós-graduação	0	1 (5,3)
**FE média**	40,4±16,3^*^	41,2±17,8^*^
FE		
ICFEr	12 (63,2)	10 (52,6)
ICFEp	4 (21,1)	5 (26,3)
ICFElr	3 (15,8)	4 (21,1)
**Mini-Mental**	27,6±2,4^*^	27,4±2,12^*^

NYHA: New York Heart Association; FE: Fração de ejeção; ICFEr: insuficiência cardíaca de fração de ejeção reduzida; ICFEp: insuficiência cardíaca de fração de ejeção preservada; ICFElr: insuficiência cardíaca de fração de ejeção levemente reduzida. *Média±desvio padrão.

### Desfecho primário

**Figura 2:**
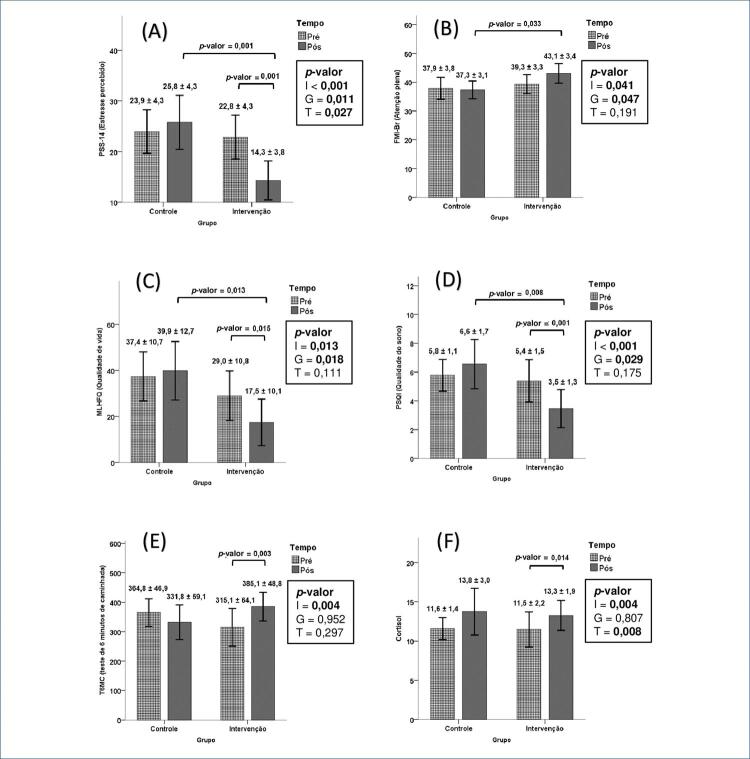
– Média e intervalo de confiança de 95% das medidas: A) Escala de estresse percebido, 14 itens – PSS-14; B) Inventário Freiburg de Mindfulness-Brasil – FMI-Br; C), Questionário de qualidade de vida de Minnesota – MLHFQ; D) Índice de qualidade do sono de Pittsburgh – PSQI; E) Teste de 6 minutos de caminhada – T6MC; F) Cortisol, todos em relação ao comportamento cruzado de grupo e tempo (p-valor dentro do quadro para I: Interação, G: Grupo, T: Tempo) e com p-valor nas chaves do resultado post-hoc < 0,05 via Bonferroni. Todas as variáveis foram medidas em pontos dos respectivos escores das escalas, exceto capacidade funcional e cortisol, que foram medidos em metros e mcg/dL, respectivamente.

Em relação ao estresse percebido (PSS-14), houve efeito significativo na interação entre tempo e grupo. Os participantes do grupo de intervenção mostraram uma diminuição no estresse percebido em comparação com o grupo de controle (-8,5 vs. +1,9; p-valor <0,001). Avaliando o resultado obtido na análise de comparações múltiplas (ACM) demonstrado na Figura 2-A, nota-se que existe diferença entre os tempos no grupo intervenção (p-valor=0,001). O PSS-14 pós foi menor (14,3) do que no período pré (22,8). Existe diferença entre os grupos no período pós (*p*-valor=0,001), o escore PSS-14 do grupo intervenção foi menor (14,3) do que o do controle (25,8).

### Desfechos secundários

Na variável atenção plena (FMI-Br) houve efeito significativo na interação. Os participantes do grupo de intervenção mostraram um aumento na atenção plena em comparação com o grupo de controle (+3,8 vs. -0,6; p-valor=0,041), conforme figura 2-B. Avaliando o resultado obtido na ACM, nota-se que existe diferença entre os grupos no período pós (p-valor=0,033). O FMI-Br do grupo intervenção foi maior (43,1) do que o controle (37,3).

Quanto à qualidade de vida (MLHFQ) houve efeito significativo na interação. Os participantes do grupo intervenção apresentaram um aumento na qualidade de vida em relação ao grupo controle (-11,5 vs.+2,5; p-valor=0,013). Avaliando o resultado obtido na ACM, nota-se que existe diferença entre os tempos no grupo intervenção (p-valor=0,015). O MLHFQ pós foi menor (17,5) do que no período pré (29,0). Existe diferença entre os grupos no período pós (p-valor=0,013), o MLHFQ do grupo intervenção foi menor (17,5) do que o do controle (39,9) conforme Figura 2-C.

Houve efeito significativo da interação na qualidade do sono (PSQI). Os participantes do grupo intervenção apresentaram um aumento na qualidade do sono em relação ao grupo controle (-1,9 vs. +0,8; p-valor<0,001). Avaliando o resultado obtido na ACM, nota-se diferença entre os tempos no grupo intervenção (p-valor=0,001). O PSQI pós foi menor (3,5) do que no período pré (5,4). Existe diferença entre os grupos no período pós (p-valor=0,008), o PSQI do grupo intervenção foi menor (3,5) do que o controle (6,6), conforme Figura 2-D.

Em relação ao T6MC houve efeito significativo na interação. Os participantes do grupo de intervenção mostraram um aumento na capacidade de exercício em comparação com o grupo de controle (+70 vs. -33; p-valor=0,004), conforme a Figura 2-E. Avaliando o resultado obtido na ACM, existe diferença entre os tempos no grupo intervenção (p-valor=0,003). O T6MC pós foi maior (385,1) do que no período pré (331,8).

Sobre o cortisol houve efeito significativo na interação. Os participantes do grupo intervenção apresentaram menor aumento do cortisol em comparação ao grupo controle (+1,8 vs. +2,2; p-valor=0,004). Avaliando o resultado obtido na ACM, existe diferença entre os tempos no grupo intervenção (p-valor=0,014). O cortisol pós foi maior (13,3) do que no período pré (11,5), porém seu aumento foi menor que no grupo controle (Figura 2-F).

Para as medidas de ansiedade traço e estado (IDATE-T e IDATE-E) e depressão (BDI-II) não houve resultados significativos, com p-valores da interação = 0,126, 0,137 e 0,151, ou seja, seu comportamento não foi influenciado pelo efeito tempo e grupo.

Nas medidas PCR e NT-proBNP, não houve efeitos significativos em nenhum dos fatores, interação (PCR p-valor=0,098; NT-proBNP p-valor=0,538), grupo (PCR p-valor=0,561; NT-proBNP p-valor= 0,302) e tempo (PCR p-valor=0,551; NT-proBNP p-valor=0,528). Observou-se, também, grande variabilidade nestas medidas coletadas.

Para a medida VHS não houve resultados significativos na interação (p-valor=0,444), porém houve diferenças entre os grupos (p-valor=0,026). Observou-se grande variabilidade nessa medida.

A Figura Central resume os principais resultados.

## Discussão

Neste estudo foi desenvolvido pela primeira vez um programa para redução do estresse em pacientes com IC, combinando ferramentas de enfrentamento, atenção plena e propósito. Não foram observados efeitos adversos importantes durante a intervenção.

Em relação ao desfecho primário, houve melhora significativa do estresse percebido no grupo da intervenção (p-valor<0,001), o que foi observado em estudos prévios utilizando treinamento de redução do estresse para pacientes com doença coronariana.^33,34^

Neste último, também foi observada melhora da atenção plena, desfecho alcançado nos pacientes submetidos à intervenção no presente estudo (p-valor<0,041), porém ainda não identificado em ensaios clínicos randomizados e controlados com pacientes portadores de IC.^31^

Em relação aos demais desfechos secundários, houve melhora significativa da qualidade de vida dos pacientes (p-valor<0,013), o que é associado a prognóstico, mortalidade e hospitalização.^35^ Tal resultado foi demonstrado por apenas dois estudos prévios envolvendo a prática de atenção plena em portadores de IC,^29,36^ sendo um deles estudo piloto, conforme apontado em revisão sistemática prévia.^37^ Há de se salientar que esta variável é considerada mais importante do que a longevidade com a progressão da IC.^38^

Os distúrbios do sono são muito prevalentes em pacientes portadores de IC^5,39-43^e foi observada melhora da qualidade do sono no grupo da intervenção (p-valor<0,001), com piora de tal variável no grupo controle.

A intervenção também melhorou significativamente a capacidade para o exercício (T6MC) em concordância com estudos prévios que utilizaram ferramentas de gestão do estresse.^32,44^ Ainda assim, nenhum estudo havia demonstrado tal desfecho após intervenção baseada em atenção plena em pacientes com IC (31). Vale ressaltar que houve piora de tal desfecho no grupo controle e que valores do T6MC têm sido associados a morbidade e mortalidade em pacientes com IC.^45,46^

Os pacientes do grupo controle apresentaram piora nos escores das variáveis citadas, demonstrando que a intervenção não apenas trouxe melhorias como impediu a progressão do estresse percebido e a deterioração da qualidade de vida, da atenção plena, da qualidade do sono e da capacidade funcional com a progressão da IC.

Foi observada tendência a melhora não significativa nos desfechos ansiedade e depressão. Estudos futuros que avaliem intervenções comportamentais destinadas à melhora destas variáveis talvez necessitem de maior duração para demonstrar efeitos significativos, conforme proposto previamente.^47^

Sobre os biomarcadores PCR, VHS, NT-proBNP e cortisol, houve resultado significativo apenas em relação ao cortisol, demonstrando tendência ao aumento desta variável ao longo do tempo em ambos os grupos, com variação média maior no grupo controle em relação à intervenção (2,2 vs. 1,8, p-valor<0,04).

A secreção de cortisol aumenta em resposta ao estresse e este biomarcador é fator independente (p-valor=0,02) para predizer eventos cardíacos em portadores de IC.^48,49^ Estudo prévio, controlado e não randomizado, avaliando 45 pessoas saudáveis, das quais 30 foram submetidas a um programa de redução do estresse, mostrou redução significativa do cortisol salivar após 4 semanas de intervenção.^50^ Porém, em relação a pacientes portadores de IC, não houve alteração no nível de cortisol sérico desta população após intervenção baseada em meditação.^44^

Estudo brasileiro avaliando o impacto de um programa de meditação de 12 semanas nos níveis de outro hormônio relacionado ao estresse (norepinefrina) demonstrou melhora significativa na redução dos níveis deste marcador no grupo da intervenção (p-valor=0,008) em relação ao controle, com p-valor=0,009.^51^

A diferença entre esses resultados, comparando as populações estudadas (68,4% em classe funcional II de NYHA no presente estudo *vs.* 84,2% em classe funcional I de NYHA no estudo prévio), sugere que, com a evolução da doença, portadores de IC possam apresentar status autonômico basal que não seria alterado pelas práticas meditativas. Outra hipótese é que seria necessário maior tempo de exposição à intervenção para haver melhora significativa em marcadores do estresse, conforme sugerido.^12^

Estudos futuros destinados aos portadores de IC sintomática, utilizando protocolos de intervenção com maior duração, serão necessários para avaliar o impacto de programas de redução do estresse nos desfechos clínicos e biomarcadores analisados.

A limitação do estudo foi a perda de parte da amostra de pacientes. Tal fator pode ter representado ausência de significância estatística em alguns desfechos analisados. A principal causa apontada para saída do estudo foi a distância dos domicílios ao centro de intervenção. Tal resultado salienta a necessidade de se estabelecer um limite de distância dos domicílios dos pacientes em relação ao centro de intervenção.

## Conclusão

A presente pesquisa demonstrou que o Programa de Redução do Estresse, Meditação e Atenção plena levou à redução significativa do Estresse Percebido em pacientes portadores de IC em centros especializados. Melhora significativa na qualidade de vida, na atenção plena, na qualidade do sono e na capacidade funcional foi observada nestes pacientes. Foi observada tendência a melhora não significativa nos desfechos ansiedade e depressão. Observou-se menor aumento do cortisol nos indivíduos submetidos ao programa em comparação ao controle e não se observou alteração nos marcadores inflamatórios e no NT-proBNP. A presente pesquisa demonstrou potencial de fornecer opção de terapia comportamental que possa contribuir para a melhora deste relevante problema de saúde pública, por meio de um método efetivo, simples e seguro.
