# Supplementary material for: Impacto de um Programa de Redução do Estresse, Meditação e Mindfulness em Pacientes com Insuficiência Cardíaca Crônica: Um Ensaio Clínico Randomizado
Source: Arq Bras Cardiol. 2023 Oct 16;120(10):e20220768. [Article in Portuguese] doi: 10.36660/abc.20220768 (PMC10586813; doi:10.36660/abc.20220768)
Supplement: Translation

# Translation: Impact of a Stress Reduction, Meditation, and Mindfulness Program in Patients with Chronic Heart Failure: A Randomized Controlled Trial

**Authors:** Vaisnava Nogueira Cavalcante, Evandro Tinoco Mesquita, Ana Carla Dantas Cavalcanti, Jacqueline Sampaio dos Santos Miranda, Paola Pugian Jardim, Glaucio Martins da Silva Bandeira, Lais Marcelle Rufino Guimarães, Isabella Christina Diniz de Lemos Venâncio, Nathalia Manoela Condeixa Correa, Angela Maria Rodrigues Dantas, João Carlos Tress, Ana Catarina Romano, Fabiana Bergamin Muccillo, Marina Einstoss Barbosa Siqueira, Glaucia Cristina Andrade Vieira

**Affiliations:** 1 Brasil Universidade Federal Fluminense, Niterói, RJ – Brasil; 2 Brasil Universidade Federal Fluminense - Departamento de Fundamentos de Enfermagem e Administração, Rio de Janeiro, RJ – Brasil; 3 Brasil Instituto Nacional de Cardiologia, Rio de Janeiro, RJ – Brasil; 4 Brasil Complexo Hospitalar de Niterói (CHN), Niterói, RJ – Brasil

**Keywords:** Stress, Psychological, Medicine, Behavioral, Mindfulness, Meditation, Heart Failure

## Abstract

**Background:**

Heart Failure is a significant public health problem leading to a high burden of physical and psychological symptoms despite optimized therapy.

**Objective:**

To evaluate primarily the impact of a Stress Reduction, Meditation, and Mindfulness Program on stress reduction of patients with Heart Failure.

**Methods:**

A randomized and controlled clinical trial assessed the effect of a stress reduction program compared to conventional multidisciplinary care in two specialized centers in Brazil. The data collection period took place between April and October 2019. Thirty-eight patients were included and allocated to the intervention or control groups. The intervention took place over 8 weeks. The protocol assessed the scales of perceived stress, depression, quality of life, anxiety, mindfulness, quality of sleep, a 6-minute walk test, and biomarkers analyzed by a blinded team, considering a p-value <0.05 statistically significant.

**Results:**

The intervention resulted in a significant reduction in perceived stress from 22.8 ± 4.3 to 14.3 ± 3.8 points in the perceived stress scale-14 items in the intervention group vs. 23.9 ± 4.3 to 25.8 ± 5.4 in the control group (p-value<0.001). A significant improvement in quality of life (p-value=0.013), mindfulness (p-value=0.041), quality of sleep (p-value<0.001), and the 6-minute walk test (p-value=0.004) was also observed in the group under intervention in comparison with the control.

**Conclusion:**

The Stress Reduction, Meditation, and Mindfulness Program effectively reduced perceived stress and improved clinical outcomes in patients with chronic Heart Failure.

## Introduction

Heart failure (HF) is an important public health problem that affects 26 million people worldwide, with a projection for an increase in prevalence with population aging and prolonged survival.^1,2^ It is responsible for a high burden on the health system due to high morbidity, mortality, hospitalization, and costs.^3,4^

Studies point out a high prevalence of emotional disorders in patients with HF, which contributes to worsening morbidity, quality of life, and mortality.^5,6^ Psychosocial stress and its components are associated with increased neurohormonal activation and unfavorable outcomes.^7-10^

Patients with HF live with increased psychosocial and existential stress, explained by the progressive and limiting disease, which changes work status and socialization and impacts their quality of life.^10^

A prospective cohort evaluated 6985 individuals and showed that a low sense of purpose is significantly associated with all-cause mortality. It shows the need for research that assesses interventions that promote a meaningful life to improve health outcomes.^11^

Several scientific evidence regarding brain-heart interactions have led to the growth of the research field in behavioral cardiology.^12^ Studies have exemplified the connection between human psychosocial stress conditions and increased proinflammatory activity.^13-16^ The induction of stress leads to activation of the autonomic nervous system (ANS) and increased production of inflammatory cytokines.^17^

HF is characterized by a chronic inflammatory state and ANS imbalance, contributing to its progression.^18^ The association between depression and HF could thus contribute to increased mortality risk in these patients.^19^

It has been shown that, through emotions and thoughts, the ANS plays a relevant role in the brain-heart connection.^12^ Mind-body practices can benefit endothelial, neuroendocrine, and immune functioning by acting through the ANS.^20-22^

Mindfulness is the ability to pay attention to the present moment intentionally and without judgment.^23^ Mindfulness interventions are effective in reducing depression, sympathetic activity, anxiety, psychosocial stress, and pain and in improving functional capacity and quality of life.^24-29^ High levels of mindfulness are related to lower anxiety levels in patients with HF.^30^

Few studies have been developed evaluating the impact of interventions based on Mindfulness in patients with HF. Therefore, it is discussed the need to carry out more studies with methodological rigor and assess the impact of these interventions on the perceived stress, functional capacity, and the levels of mindfulness of these individuals.^31^

Consequently, the present study aimed primarily to evaluate the impact of the Stress Reduction, Meditation, and Mindfulness Program on HF patients’ perceived stress in specialized centers. Anxiety, depression, mindfulness, quality of life, quality of sleep, functional capacity (6-minute walk test), an inflammatory panel including erythrocyte sedimentation rate (ESR) and c-reactive protein (CRP), and neurohormonal panel (cortisol and NT-proBNP) were evaluated secondarily.

## Methods

### Study design

A randomized controlled trial (RCT) was carried out in four parallel and distinct groups. The intervention groups (I-CICCV and I-INC) participated in the 8-week stress reduction program. The control groups (C-CICCV and C-INC) received conventional multidisciplinary care from the Clínica de Insuficiência Cardíaca Coração Valente (CICCV)/UFF and the Department of Heart Failure and Transplantation of the Instituto Nacional de Cardiologia (INC), from where patients in the groups were recruited.

### Participants

Outpatients aged over 18 years, diagnosed with HF by the Framingham and/or Boston criteria; NYHA classification I-II; Mini-Mental > 16; adherent to at least 80% of the meetings; who were literate or who have a literate caretaker; who agreed to participate in the proposed activities and to respond to the assessment questionnaires; who had a sound device to listen to the audios with daily practices were included in the study.

Patients who had concomitant participation in another study/therapy involving mind-body intervention in the last month; with recent clinical decompensation with hospitalization or change in drug prescription in the previous month; who had surgery or percutaneous intervention planned for the next year; with a history of acute coronary syndrome or acute myocardial infarction in the last two months; with clinically significant valvular heart disease; with acute myocarditis; with musculoskeletal conditions that prevented the performance of functional tests such as the 6-minute walk test (6MWT) were excluded.

### Intervention

The Stress Reduction Meditation and Mindfulness Program (PREMA) is based on the tripod: coping, mindfulness, and *dharma* (Sanskrit term for purpose in life) tools. The sessions took place over 8 weekly group meetings, each lasting 2 hours, consisting of 4 moments:

1. Introduction.
2. Theme presentation.
3. Self-awareness questions.
4. Guided practice.

The topics covered were stress, the Mind, resilience, emotional self-regulation, acceptance, self-efficacy, gratitude, social connections, and purpose. Each session addressed a coping tool related to the theme.

The mindfulness practices addressed throughout the sessions were mindful breathing, mindful eating, mindful inventory of negative and positive emotions, compassion meditation, mindful walking, and reciprocity meditation.

The sessions took place between May and July 2019 at the premises of the CICCV (for I-CICCV) and the Instituto Nacional de Cardiologia (for I-INC), and the data was collected from April to October 2019. The program was facilitated by the leading study researcher, a cardiologist with 23 years of experience in meditation and bhakti yoga, an internship in a hospital focusing on mind-body-spirit therapies (Bhaktivedanta Hospital – Mumbai/India), training in SMART® (Stress Management and Resilience Training) and Mindfulness programs, both from Case Western Reserve University and certification in yoga from VVY School.

Support materials such as a handout and an audio CD for 45 minutes of daily practice were provided. Adherence to weekly tasks was observed through patient reports, meditation diary, and telemonitoring.

The primary outcome assessed was perceived stress, and secondary outcomes were scores for anxiety, depression, mindfulness, quality of life, quality of sleep, functional capacity (6MWT), inflammatory panel (ESR, CRP), and neurohormonal panel (cortisol and NT-proBNP).

### Sample size

The sample was calculated based on a previous study that used the ‘perceived stress’ outcome, in which the following score values were used: before the intervention (control group=19.1/SD±8.9; intervention=25.8/SD±4.6) and after the intervention (control group=20.5 /SD±10.3; intervention group=20.2/SD±5.6).^32^The final sample consisted of 36 patients, 18 for the intervention group and 18 for the control group, after considering a difference of 0,3 points, 95% confidence, 20% loss, and 80% power.

### Randomization

Randomization was performed through the website http://randomization.com, considering a 20% loss. A professional external to the research group was responsible for the list generated for patient allocation. The allocation ratio was 1:1 for the intervention or control groups.

As it is a behavioral intervention, it was not possible to blind the intervention facilitator or the patients. However, there was blinding concerning the evaluation of the patients, which was carried out by a group of investigators responsible for collecting data and storing the results of the questionnaires, functional tests, and biomarkers.

The research team consisted of 1 evaluation group, 1 intervention group, 1 control follow-up group, and 1 randomization group.

### Evaluation

The pre-intervention assessment took place one week before the beginning of the intervention. It consisted of a face-to-face consultation with the multidisciplinary team of the specialized clinics.

At the time of the face-to-face consultation, the patient responded to the following data collection instruments: 1) Mini-mental state examination (Mini-Mental); 2) Perceived stress scale, 14 items (PSS-14); 3) Beck depression inventory, second version (BDI-II); 4) Minnesota living with HF questionnaire (MLHFQ); State-trait anxiety inventory (STAI) – STAI-T (trace) and STAI-E (state); Pittsburgh sleep quality index (PSQI); and the Brazilian version of Freiburg Mindfulness inventory (FMI-Br).

The PSS-14 is a self-report scale measuring perceived stress, consisting of 14 items with scores ranging from 0 to 56. Higher scores indicate worse stress levels. The STAI trait or state is also a self-report scale with 20 items, with scores ranging from 20 to 80. High scores on their respective scales mean greater trait or state anxiety. The BDI-II is a self-assessment scale comprising 21 items with scores ranging from 0 to 63. Higher scores indicate more severe depression.

The MLHFQ is a 21-item scale with a global score ranging from 0 to 105, with a lower score reflecting better quality of life. The PSQI assesses 7 sleep components with a global score of 0 to 21. Higher scores indicate worse sleep quality. The FMI-Br identifies the frequency with which the person experiences mindfulness-related behaviors. The score ranges from 14 to 56 points. Higher scores indicate the best perception of mindfulness.

The 6MWT was also performed on this day by physiotherapy professionals who used the American Thoracic Society guideline, as well as blood collection for the analysis of inflammatory and neurohormonal panels by the INC laboratory.

The post-intervention evaluation took place one week after the end of the intervention, including the initial protocols above. The control group received the usual multidisciplinary care of HF clinics. As these are research centers specializing in HF, patients were guaranteed continuity of care.

A pilot study was carried out with 3 patients from the CICCV clinic external to the study, before the intervention itself, to evaluate the program’s feasibility and adjust the intervention methodology.

### Statistical analysis

SPSS software version 24 was used to create the graphs and analyze the data. The mean and 95% confidence interval were used to summarize the data. The data summaries were arranged in graphs that present the combinations between the two categories of time and the two categories of the group, totaling four bars.

Continuous variables were described by mean and standard deviation. Categorical variables were described with simple frequencies and percentages. The normality test to confirm the normal distribution was the Shapiro-Wilk.

The generalized linear model (GLM) methodology for repeated measures was applied to verify the effects of interaction, time, and group. If there were significant results in the effects, a post-test was applied using the correction via Bonferroni to compare which means differ from each other, and a *p*-value <0.05 was adopted as the significance level in the study.

### Ethical aspects

This research was approved by the Ethics and Research Committee of the Faculty of Medicine of the Hospital Universitário Antônio Pedro, Report 3,224,212, by the Ethics and Research Committee of the INC, Report 3,339,599, and registered in the Brazilian Registry of Clinical Trials, under number RBR-7pzcyk.

## Results

Two hundred twenty-one patients were evaluated for inclusion in the study using a medical record search. Among these, 133 were considered eligible, with 101 patients being approached and the others not because they did not have updated telephone contact. A total of 38 patients agreed to participate in the research and were randomized to intervention and control groups. Among 38 patients, 32 adhered to and completed the assessment protocols, 13 in the intervention group (I-CICCV=9 and I-INC=4) and 19 in the control group (C-CICCV=10 and C-INC=9). Figure 3 illustrates the selection and enrollment of patients.

The sample was composed predominantly of males, self-declared brown race, NYHA II, having completed high school, and having heart failure with reduced ejection fraction. Table 1 illustrates the sociodemographic baseline characteristics of the initial sample of 38 patients:

**Table 1:** – Sociodemographic and clinical characteristics of the intervention (n=19) x control (n=19) groups. Brazil, 2019

Variables	Intervention (n=19)	Control (n=19)
Age (years)	58.0±12.8^*^	54.3±9.8^*^
**Gender**		
Male	12 (63.2)	13 (68.4)
Female	7 (36.8)	6 (31.6)
**Self-declared race**		
White	6 (31.6)	3 (15.8)
Black	4 (21.1)	4 (21.1)
Brown	9 (47.4)	12 (63.2)
**NYHA Class**		
I	7 (36.8)	5 (26.3)
II	12 (63.2)	14 (73.7)
**Complete Education**		
Elementary School	10 (52.6)	4 (21.1)
High school	9 (47.4)	14 (73.7)
Postgraduate studies	0	1 (5.3)
**Average EF**	40.4±16.3^*^	41.2±17.8^*^
EF		
HFrEF	12 (63.2)	10 (52.6)
HFpEF	4 (21.1)	5 (26.3)
HFmrEF	3 (15.8)	4 (21.1)
**Mini-Mental**	27.6±2.4^*^	27.4±2.12^*^

NYHA: New York Heart Association; EF: ejection fraction; HFrEF: heart failure with reduced ejection fraction; HFpEF: heart failure with preserved ejection Fraction; HFmrEF: heart failure with mildly reduced ejection fraction. *Mean ± standard deviation.

### Primary outcome

Regarding perceived stress (PSS-14), there was a significant effect on the interaction between time and group. Participants in the intervention group showed decreased perceived stress compared to the control group (-8.5 vs. +1.9; p-value<0.001). While evaluating the result obtained in the analysis of multiple comparisons (AMC) shown in Figure 2-A, it is noted that there is a difference between the times in the intervention group (p-value=0.001). The post-PSS-14 was lower (14.3) than the pre-period (22.8). There is a difference between the groups in the post-period (p -value=0.001); the PSS-14 score of the intervention group was lower (14.3) than the control (25.8).

**Figure 1:** – Selection process flowchart of the participants for the randomized controlled trial.

**Figure 2:** – Mean and 95% confidence interval of measurements: A) Perceived stress scale, 14 items – PSS-14; B) Freiburg Mindfulness Inventory - Brazil – FMI-Br; C) Minnesota living with heart failure questionnaire – MLHFQ; D) Pittsburgh sleep quality index – PSQI; E) 6-minute walk test – T6MC; F) Cortisol, all concerning cross-group behavior and time (p-value within the frame for I: Interaction, G: Group, T: Time) and with a p-value of the in post-hoc results < 0.05 via Bonferroni on the brackets. All the variables were measured in points of the respective scale scores, except for functional capacity and cortisol, measured in meters and mcg/dL, respectively.

### Secondary outcome

Regarding mindfulness (FMI-Br), there was a significant effect on the interaction. Participants in the intervention group showed increased mindfulness compared to the control group (+3,8 vs. -0,6; p-value=0,041), as observed in Figure 2-B. While evaluating the result obtained in the AMC, there is a difference between the groups in the post-period (p-value=0.033). The FMI-Br of the intervention group was higher (43.1) than the control (37.3).

For the quality of life (MLHFQ), there was a significant effect on the interaction. Participants in the intervention group showed an increase in quality of life compared to the control group (-11,5 vs. +2,5; p-value=0,013). While evaluating the result obtained in the AMC, there is a difference between the times in the intervention group (p-value=0.015). The post-MLHFQ was lower (17.5) than the pre-period (29.0). There is a difference between the groups in the post-period (p-value=0.013); the MLHFQ of the intervention group was lower (17.5) than the control (39.9), as shown in Figure 2-C.

There was a significant effect of interaction on sleep quality (PSQI). Participants in the intervention group showed an increase in sleep quality compared to the control group (-1,9 vs. +0,8; p-value<0.001). While evaluating the result obtained in the AMC, there is a difference between the times in the intervention group (p -value=0.001). The post-PSQI was lower (3.5) than in the pre-period (5.4). There is a difference between the groups in the post-period (p -value=0.008); the PSQI of the intervention group was lower (3.5) than the control group (6.6), as shown in Figure 2-D.

Regarding the 6MWT, there was a significant effect on the interaction. Participants in the intervention group showed increased exercise capacity compared to the control group (+70 vs. -33; p-value=0.004), as shown in Figure 2-E. While evaluating the result obtained in the AMC, there is a difference between the times in the intervention group (p-value=0.003). The post-6MWT was higher (385.1) than in the pre-period (331.8).

For the cortisol measure, there was a significant effect on the interaction. Participants in the intervention group showed a lower increase in cortisol compared to the control group (+1,8 vs. +2,2; p-value=0.004). While evaluating the result obtained in the AMC, there is a difference between the times in the intervention group (p-value=0.014). The post-cortisol was higher (13.3) than in the pre-period (11.5), but its increase was lower than in the control group (Figure 2-F).

For measures of trait and state anxiety (STAI-T and STAI-E) and depression (BDI-II), it was not possible to detect significant results, with p-values of interaction = 0.126, 0.137, and 0.151; in other words, the behavior of these measures was not influenced by the time and group effect.

In the CRP and NT-proBNP measurements, it was not possible to detect significant effects in any of the factors, interaction (CRP p-value=0.098; NT-proBNP p-value=0,538), group (CRP p-value=0.561; NT-proBNP p-value= 0,302) and time (CRP p-value=0.55; NT-proBNP p-value=0,528). It was also observed great variability in the collected measures.

For the ESR measure, it was not possible to detect significant results in the interaction (p-value=0.444). However, there were differences between the groups (p-value=0.026). It should be noted that there is great variability in this measure.

The Central Illustration resumes the main findings of this study.

## Discussion

In this study, a program was developed for the first time to reduce perceived stress in HF patients, combining coping, mindfulness, and purpose in life tools. No serious adverse events were observed during the intervention.

Regarding the primary outcome, there was a significant improvement in perceived stress in the intervention group (p-value<0.001), observed in previous studies using stress management training for patients with coronary heart disease.^33, 34^

In the latter, a significant improvement was also observed in the variable mindfulness, a secondary outcome achieved in patients under intervention in the present study (p-value <0.041) not yet identified in RCTs assessing patients with HF.^31^

Regarding the other secondary outcomes, there was a significant improvement in the patients’ quality of life (p -value<0.013), which was associated with prognosis, mortality, and hospitalization.^35^ This result was demonstrated by only two previous studies involving mindfulness practice in patients with HF, ^29,36^ one of which was a pilot study, as pointed out in a previous systematic review.^37^ It should be noted that this variable is considered more important than longevity with the progression of HF.^38^

Sleep disorders are prevalent in patients with HF.^5,39-43^ and an improvement in sleep quality was observed in the intervention group (p-value<0.001), with a worsening of this variable in the control group.

The intervention also significantly improved the exercise capacity (6MWT), in agreement with previous studies that used stress management tools.^32,44^ Still, no RCT had demonstrated such an outcome using a mindfulness-based intervention in patients with HF.^31^ It is essential to point out that there was a worsening of this outcome in the control group and that 6MWT values have been associated with morbidity and mortality in patients with HF.^45,46^

The control group presented a worsening in the scores of the variables above, showing that the intervention not only brought improvements but also prevented the progression of perceived stress and deterioration of life quality, Mindfulness, sleep quality, and functional capacity with the progression of HF.

A trend towards non-significant improvement was observed in anxiety and depression outcomes. Future studies that evaluate behavioral interventions to improve these variables may need to last longer to demonstrate significant effects, as proposed in a previous study.^47^

Regarding the biomarkers CRP, ESR, NT-proBNP, and cortisol, a significant result was found only concerning cortisol, demonstrating a tendency for this variable to increase over time in both groups, with a wider average variation in the control group in relation to the intervention (2.2 vs. 1.8, p-value<0.04).

Cortisol secretion increases in response to stress, and this biomarker is an independent factor (p-value=0.02) in predicting cardiac events in patients with HF.^48,49^ A previous, controlled, and non-randomized study evaluating 45 healthy people, of which 30 were submitted to a stress reduction program, showed a significant reduction in salivary cortisol after 4 weeks of intervention.^50^ However, concerning patients with HF, no alteration was observed in this population’s serum cortisol level after intervention based on meditation.^44^

A Brazilian study evaluating the impact of a 12-week meditation program on the levels of another stress-related hormone (norepinephrine) demonstrated a significant improvement in the reduction of levels of this marker in the intervention group (p-value=0.008) compared to the control, with a p-value=0.009.^51^

The difference between these results comparing the populations studied (68.4% in NYHA functional class II in the present study *vs.* 84.2% in NYHA functional class I in the 2005 study) suggests that, as the disease progresses, patients with HF may present a basal autonomic status that meditative practices would not alter. Another hypothesis is that it would require a longer time of exposure to the intervention to observe a significant improvement in stress-related markers, as previously suggested.^12^

Future studies aimed at patients with symptomatic HF, using longer intervention protocols, will be necessary to assess the impact of stress reduction programs on the clinical outcomes and biomarkers analyzed. The study’s limitation was the loss of part of the sample of patients. This factor may have represented the absence of statistical significance in some of the outcomes analyzed. The main reason given by the patients for leaving the study was the distance from their homes to the intervention center. This result highlights the need to establish a distance limit between the patients’ homes and the intervention center.

## Conclusion

The present research demonstrated that the Stress Reduction, Meditation, and Mindfulness Program significantly reduced perceived stress in patients with HF in specialized centers. A significant improvement was also observed in these patients’ quality of life, mindfulness, quality of sleep, and functional capacity. A trend towards non-significant improvement was observed in anxiety and depression outcomes. A smaller increase in cortisol was observed in individuals submitted to the program compared to the control, and no significant changes were observed in inflammatory markers and NT-proBNP. The present research demonstrated the potential to provide a behavioral therapy option to improve this relevant public health problem through an effective, simple, and safe method.
